# Post-mortem infant-directed behaviours in wild Guinea baboons

**DOI:** 10.1007/s10329-026-01279-8

**Published:** 2026-07-26

**Authors:** Anaïs Avilés de Diego, Federica Dal Pesco, Julia Fischer

**Affiliations:** 1 Department for Primate Cognition, Georg-August-Universität, Göttingen, Germany; 2 Cognitive Ethology Laboratory, German Primate Centre, GmbH, Kellnerweg 4, 37077 Göttingen, Germany

**Keywords:** Dead infant, Mother-infant bond, *Papio papio*, Post-mortem behaviours, Thanatology

## Abstract

The growing field of comparative thanatology aims to shed light on how and why the understanding of death evolved. Observations across different nonhuman primate species have reported care-taking behaviour toward dead infants, but also cannibalism. Various hypotheses have been put forward to explain these behaviours, ranging from responses to infantile cues to an understanding of death. To aid comparative analyses and future tests of these hypotheses, we report behaviours toward dead infants in a wild population of Guinea baboons (*Papio papio*) living in the Niokolo-Koba National Park, Senegal. During 12 years of field observations (2014–2025), we recorded 64 infant deaths within the first year of life. In 22 of these cases, mothers, but occasionally also other group members, carried, protected, groomed, and dragged dead infants. In 6 cases, cannibalism occurred. None of the mothers expressed any signs of emotional distress in response to infant death. We suggest that no concept of death in Guinea baboons needs to be invoked to explain the observed behaviours. Instead, post-mortem caretaking behaviours can be viewed as the by-product of selection for infant care, which includes not abandoning an infant that might temporarily be unresponsive. The lack of infant responses to maternal behaviour, together with the disintegration of the corpse, may drive a shift from perceiving the infants as an object that elicits caretaking to one that is either abandoned or, in some cases, comes to resemble food, ultimately facilitating cannibalism.

**Supplementary Information:**

The online version contains supplementary material available at https://doi.org/10.1007/s10329-026-01279-8.

## Introduction

Over the last two decades, increasing interest in animal cognition, emotion, and sentience, together with a growing body of systematic field and captive observations of behaviours directed towards dying or dead conspecifics (hereafter “post-mortem behaviours”), has fuelled renewed interest in whether nonhuman animals show at least a rudimentary sensitivity to death-related cues. A related question is whether such responses may illuminate the evolutionary roots of human concepts of death, which involve the understanding of the irrevocable end of life (Anderson 2016, 2017; Gonçalves and Carvalho 2019; Monsó and Osuna-Mascaró 2021). Comparative thanatology has thus emerged as an explicitly interdisciplinary framework for examining death and dying across taxa, encompassing the behavioural, physiological, cognitive, and social responses elicited by dead or dying conspecifics (Anderson 2016; Gonçalves and Biro 2018; Gonçalves and Carvalho 2019). This work now spans a broad comparative spectrum, from experimentally tractable insect systems—such as necrophoresis in ants, where corpse removal can be triggered by fatty-acid cues such as oleic acid or by the loss of “life signals” (Choe et al. 2009; Wilson et al. 1958)—to birds, where corvids gather around dead conspecifics and use these events to learn about danger (Swift and Marzluff 2015, 2018), to large-brained mammals including cetaceans and elephants (Bearzi et al. 2018; Goldenberg and Wittemyer 2020; McComb et al. 2006).

Among primates, post-mortem responses range from infant corpse carrying (hereafter “ICC”) to grooming and even cannibalisation, suggesting that death-related responses vary along a continuum from relatively simple cue-driven reactions to more complex and varied behaviours (Anderson et al. 2018; Gonçalves and Carvalho 2019; Lonsdorf et al. 2020; Soldati et al. 2022). Studying the loss of relatives and social partners across taxa may therefore help reconstruct the evolutionary pathways underlying both immediate responses to death and the more complex social consequences of bereavement. Post-mortem behaviours have been observed in a wide variety of primates (e.g., chimpanzees, *Pan troglodytes*: Bersacola et al. 2025; chacma baboons, *Papio ursinus*: Carter et al. 2020; Sumatran orangutans, *Pongo abelii*: Dellatore et al. 2009; northern muriquis, *Brachyteles hypoxanthus*: Freire Filho et al. 2022; Indian langurs, *Presbytis entellus*: Jay 1962; mountain gorillas, *Gorilla beringei beringei*: Warren and Williamson 2004). In these studies, the most frequently observed behaviours are inspection, protection, grooming, and carrying directed by mothers towards their dead infants, although other conspecifics may occasionally engage with them as well (Carter et al. 2020; Merz 1978). Whereas some mothers abandon their deceased infants soon after they die, others continue interacting with them for extended periods of time (e.g., > 10 days; Fashing et al. 2011).

Infant corpse carrying is the most prominent post-mortem behaviour in primates (reviewed in Fernández-Fueyo et al. 2021; Gonçalves and Carvalho 2019; Watson and Matsuzawa 2018). As dead infants cannot hold onto their mother’s fur, mothers may transport the infant corpse in their mouth, ventrally supported by one arm (Bersacola et al. 2025; Carter et al. 2020; Watson and Matsuzawa 2018), slung across the carrier’s back (Bersacola et al. 2025; Biro et al. 2010), or dragged (Bersacola et al. 2025; Carter et al. 2020; Gonçalves and Carvalho 2019). Carrying is energetically costly, particularly in species that forage over long daily distances (Altmann and Samuels 1992). Carrying a dead infant hinders foraging and locomotion, thereby increasing predation risks for the carrier (Gonçalves and Carvalho 2019).

Several non-mutually exclusive hypotheses have been put forward to explain the cognitive, emotional, and physiological mechanisms underlying maternal post-mortem behaviours (reviewed in Gonçalves and Carvalho 2019; Watson and Matsuzawa 2018). Some of these hypotheses imply a concept of death as the irrevocable end of life or view ICC as a form of grief (Cronin et al. 2011; Takeshita et al. 2020). Other authors do not assume a concept of death (‘unawareness hypothesis’). According to this hypothesis, mothers cannot discern if their infants are dead or perhaps temporarily unresponsive and continue to treat them as alive because it is ultimately maladaptive to abandon a non-responsive infant (Sugiyama et al. 2009). Other studies suggest that mothers respond to infant cues (Alley 1980) or other cues (e.g., fur) that elicit caretaking behaviour (Reiderman et al. 2024). Post-mortem behaviours have also been linked to the hormonal state of the mother (‘post parturient hypothesis’; e.g., Biro et al. 2010; Kaplan 1973), and are suggested to be modulated by the bond strength between the mother and the infant (‘mother-infant bond hypothesis’; e.g., Fernández-Fueyo et al. 2021; Matsuzawa 1997).

Other hypotheses concern variation among mothers in the display of post-mortem behaviours. The ‘learning-to-mother hypothesis*’* suggests that nulliparous females may interact with deceased infants of other females to gain experience in mothering (Warren and Williamson 2004; Watson and Matsuzawa 2018). The ‘parity hypothesis*’*, in contrast, proposes that multiparous mothers will display ICC behaviours for longer (Sharma et al. 2011), potentially because older mothers place greater value on investing in current offspring (Clutton-Brock 1984). Das and colleagues (2019) analysed 43 cases across 18 primate species and revealed that older mothers carried dead infants for longer. An analysis based on 104 published cases of dead infant carrying in 20 primate species (Fernández-Fueyo et al. 2021) showed that dead-infant carrying was more likely in younger mothers. However, there was considerable variation between species, and in 122 cases of ICC with known maternal age in Japanese macaques (*Macaca fuscata*), there was no difference in propensity to carry the infant between older and younger mothers (Sugiyama et al. 2009). These findings underscore the importance of potential species-specific patterns.

Cannibalism of the corpse has also been described for several primate species. On most occasions, it involves a mother eating parts of her dead infant (*filial* or *maternal cannibalism*; e.g., vervet monkey, *Chlorocebus pygerythrus*: Botting and van de Waal 2020; drills, *Mandrillus leucophaeus*: Casetta et al. 2023; Tonkean macaques, *Macaca tonkeana*: De Marco et al. 2018), but it can also be observed in conspecifics other than the mother (*conspecific necrophagy*; e.g., bonobos, *Pan paniscus*: Fowler and Hohmann 2010; white-faced capuchins, *Cebus imitator*: Kulick et al. 2021; brown capuchin monkeys, *Sapajus apella*: Trapanese et al. 2020). Cannibalism can provide relatively high-quality food (Milton 2003), but it may also promote pathogen transmission (Rudolf and Antonovics 2007). Some authors have suggested environmental stress and abnormal attachment as potential causes (Fedurek et al. 2020), while others consider it an aberrant behaviour (Dellatore et al. 2009) or simply part of a species’ behavioural repertoire (Tokuyama et al. 2017).

To add to the database on post-mortem behaviours toward infants and contribute to broader tests of these hypotheses, we here use twelve years of long-term data to report 22 opportunistic observations of care-taking behaviours and cannibalism towards dead infants (up to 12 months of age) in a wild population of Guinea baboons (*Papio papio*). Given the lack of prior information on this species, our analyses are exploratory, and we had no specific hypotheses or predictions regarding the occurrence of post-mortem behaviour. In the discussion, we will examine the findings in light of current hypotheses in the field.

## Methods

### Study species and site

We studied wild Guinea baboons living in the Niokolo-Koba National Park, in Senegal, near the facilities of the Centre de Recherche de Primatologie (CRP) Simenti (13 °01 ′34 ″N, 13 °17 ′41 ″W) (Fischer et al. 2017). Guinea baboons live in nested multi-level societies. ‘Units’ are at the base of the society and are composed of one ‘primary’ male and one to eight females and their immatures (Dal Pesco et al. 2022; Fischer et al. 2017; unpublished data). Several units group together with bachelor males to form a ‘party’, and two or more parties associate to form ‘gangs’ (Patzelt et al. 2014). Importantly, Guinea baboons show female-biased dispersal, and females may transfer at all levels of the society. The community of Guinea baboons in our study area comprises 350–400 individuals (Dal Pesco et al. 2021; Zinner et al. 2021), of which approximately 160 are habituated and identified by physical characteristics. Senegal experiences marked seasonality, with a dry season lasting from November to May and a rainy season from June to October.

### Data collection

We collected data between April 2014 and September 2025 on parties 5, 6 (later split into 6I and 6 W), 9 (later split into two parties, with party 9B remaining in our study area), and party 13. The study period encompassed more than 9,000 h of focal observations. All observers completed a reliability test based on repeated parallel observations (Dal Pesco and Fischer 2022) with an experienced observer before collecting data independently. In rare cases, trainees recorded events only under direct supervision. Observations of post-mortem infant-directed behaviours were recorded and entered into the database by 17 trained observers with varying levels of experience (mean 1.8 years; range: 48 days to > 7 years since starting in the project); usually, several observers witnessed the behaviours. We included all behaviours towards dead infants up to 1 year of age. During the study period, 68 infants died before reaching 1 year of age. In 4 of the 68 cases, we could not establish the age at death because the field station was closed due to the COVID-19 pandemic. We also had no information on whether post-mortem infant-directed behaviours occurred, leaving 64 cases for the present analysis. We recorded behavioural observations using handheld devices (Samsung Galaxy Note II GT-7100 or Gigaset GX290 smartphones) and electronic forms designed with Pendragon Software Corporation’s version 7.2.21 software (Chicago, IL, USA). We augmented our observations using photographs and video.

During the study period, the following post-mortem behaviours were observed: carrying, protecting, grooming, dragging, cannibalism, handling, and meat transfer (Table 1). The information recorded consisted of the date of the event, infant ID (i.e., corpse), the ID of the actor of the post-mortem behaviours, the type of relationship between corpse and actor ID (i.e., mother versus non-mother), the duration of post-mortem behaviours between actor ID and corpse, and type of post-mortem behaviours observed; all other potentially relevant information was recorded *ad libitum*. Furthermore, we determined the infant’s age at death and the actor’s parity (if the actor was female) from our long-term demographic database, which contains daily information on significant life-history and social events, such as births, deaths, health status, transfers, and female reproductive states. In addition, meteorological data were recorded daily, including minimum and maximum temperatures, minimum and maximum relative humidity, and precipitation.

**Table 1:** Ethogram of post-mortem behaviours that were directed to Guinea baboon infant corpses from April 2014 to September 2025

Behaviour	Definition
Carrying	The mother (or another individual) carries the corpse, ventrally supported with one arm, held in one hand, or in the mouth
Protecting	The mother (or another individual) tightly holds or grabs the corpse with one or both arms or feet, potentially intensifying when other members are in proximity, thereby limiting the latters’ access to the corpse or pulling back if they try to get hold of it.
Grooming	The mother (or another individual) gently fingers and/or mouths the fur and skin of the corpse
Dragging	The mother (or another individual) pulls the corpse across the ground by grabbing it by one of its extremities, tail, or torso.
Cannibalism	The mother (or another individual) consumes part of the corpse.
Handling	The actor touches and may lift the corpse
Meat transfer	The mother (or another individual), while consuming the corpse, tolerates taking pieces of meat by another individual (*passive meat sharing*; see Goffe and Fischer 2016).

### Determination of the duration of post-mortem behaviours with a corpse

We assessed the duration of post-mortem behaviours with a dead infant based on the number of days that the researchers observed the corpse and post-mortem behaviours. The actual duration of post-mortem behaviours could not always be accurately determined. For seven cases (case 8, 16, 17, 18, 19, 21 and 22), we were able to record the total duration of the event, as we saw the mother and the infant alive the day before the post-mortem behaviour began, and the mother without the corpse the day after the last sighting of it (Table 2, equal observed and estimated duration). For case 19, we had not seen the mother and infant on the day prior to the first sighting of the infant’s corpse, but based on the corpse’s fresh appearance, we inferred that he had likely died earlier that morning. In the remaining 15 cases, there was a gap between the last day we saw the mother with the infant alive and the first day we saw the corpse, or the last day we saw the corpse and the first day we saw the mother again without it, or both. In these cases, we counted the days on which the researchers observed the corpse (Table 2, observed duration), although the actual duration of behaviours towards the dead body may have been longer (Table 2, estimated duration). In five of these cases (1, 3, 6, 10, and 15), we had additional information (e.g., comments in our data about the corpse’s condition) that suggested a longer carrying duration than the days of actual observation, reported in the table legend under (Table 2, estimated duration).

## Results

### Infant age and causes of death

For the 64 infants that died within the first year of life and for whom we had reliable data on the age of death, we observed 22 instances of post-mortem infant-directed behaviours. In these cases, mothers – but occasionally also other group members – carried, protected, groomed, and dragged dead infants (summarised in Table 2). These events were not restricted to a particular season, with 8 cases (36%) observed during the rainy season and 14 cases (64%) during the dry season (Table 2), which roughly corresponds to the relative lengths of the rainy (42%) and dry seasons (58%). Most cases (20 out of 22) involved infants up to 6 months old (Table 2); 6 were stillbirths, 4 were less than 1 week old, 8 were between 1 week and 3 months old, and 2 were between 3 and 6 months old. The two remaining cases were infants older than 6 months; therefore, they had already transitioned to “brown” infants (for age category definitions, see Dal Pesco and Fischer 2022), with the older one dying at 226 days. The mean age at death of infants for whom we observed post-mortem infant-directed behaviours was 47.1 days (*N* = 22, range 0-226), whereas infants receiving no such behaviours were, on average, 121.2 days old (*N* = 42, range 1-359 days). For some of the infants for whom we did not observe post-mortem behaviours, it is possible that we missed such behaviours because we did not follow the group on that particular day or the next. It is also possible that the mothers simply abandoned the corpse, or it was taken by scavengers.

**Fig. 1:**
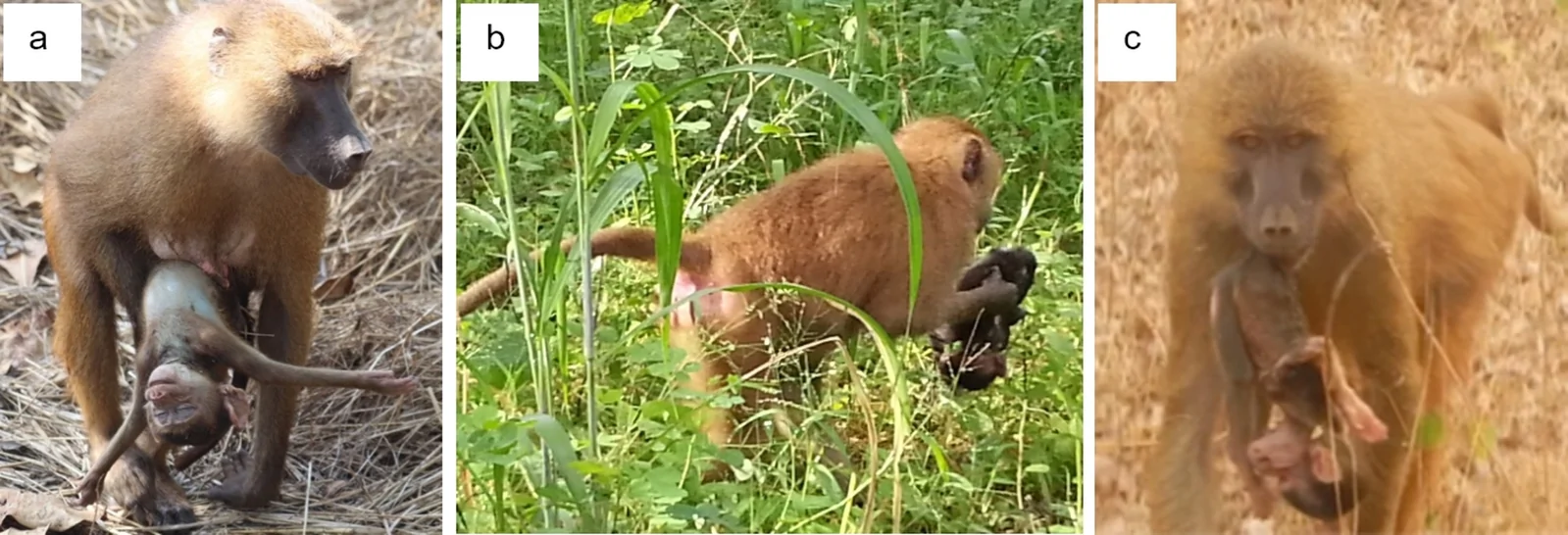
The modes of carrying a dead infant in Guinea baboons include (a) ventrally supporting the corpse with one arm (case 19), (b) holding it in one hand (case 1), and (c) in the mouth (case 5). Picture (a) taken by Anaïs Avilés de Diego, picture (b) taken by Federica Dal Pesco, and picture (c) taken by Lauriane Faraut; Cognitive Ethology Laboratory, German Primate Centre

**Fig. 2:**
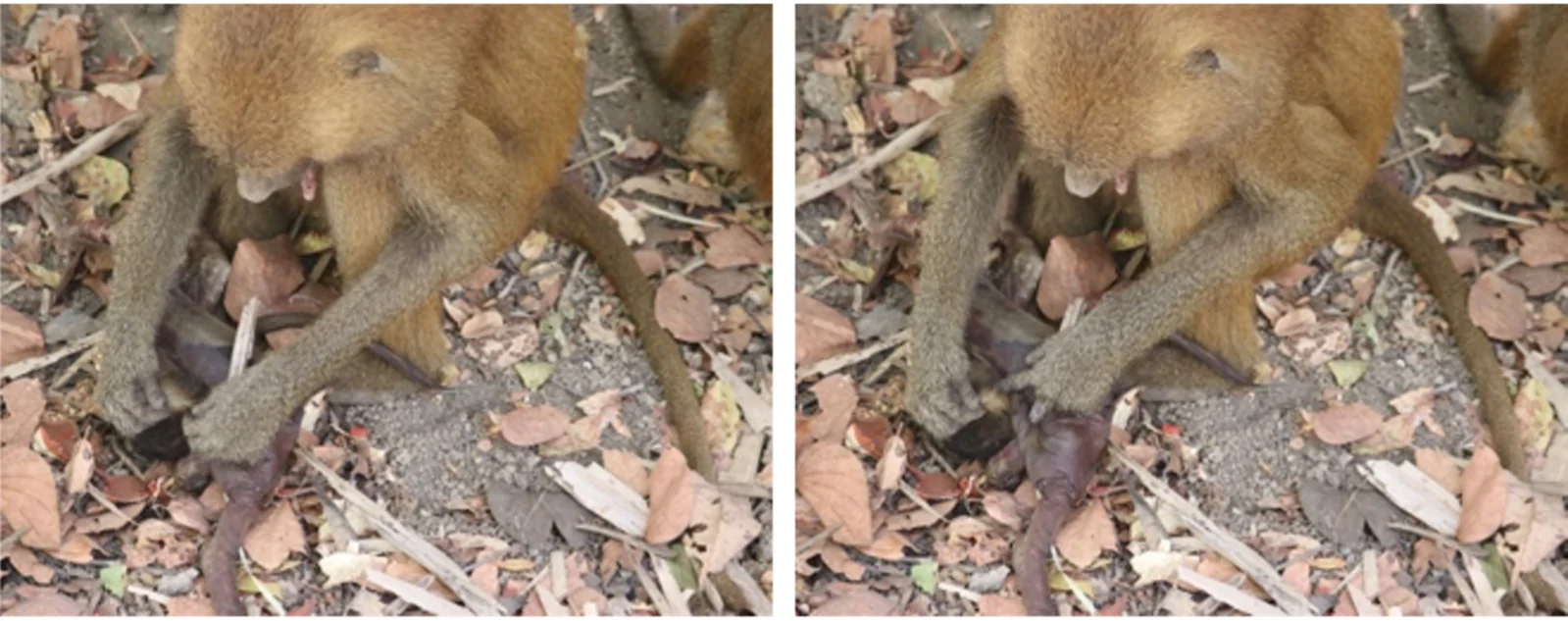
Dead infant being groomed by his mother (case 10). Pictures taken by Anaïs Avilés de Diego; Cognitive Ethology Laboratory, German Primate Centre

**Fig. 3:**
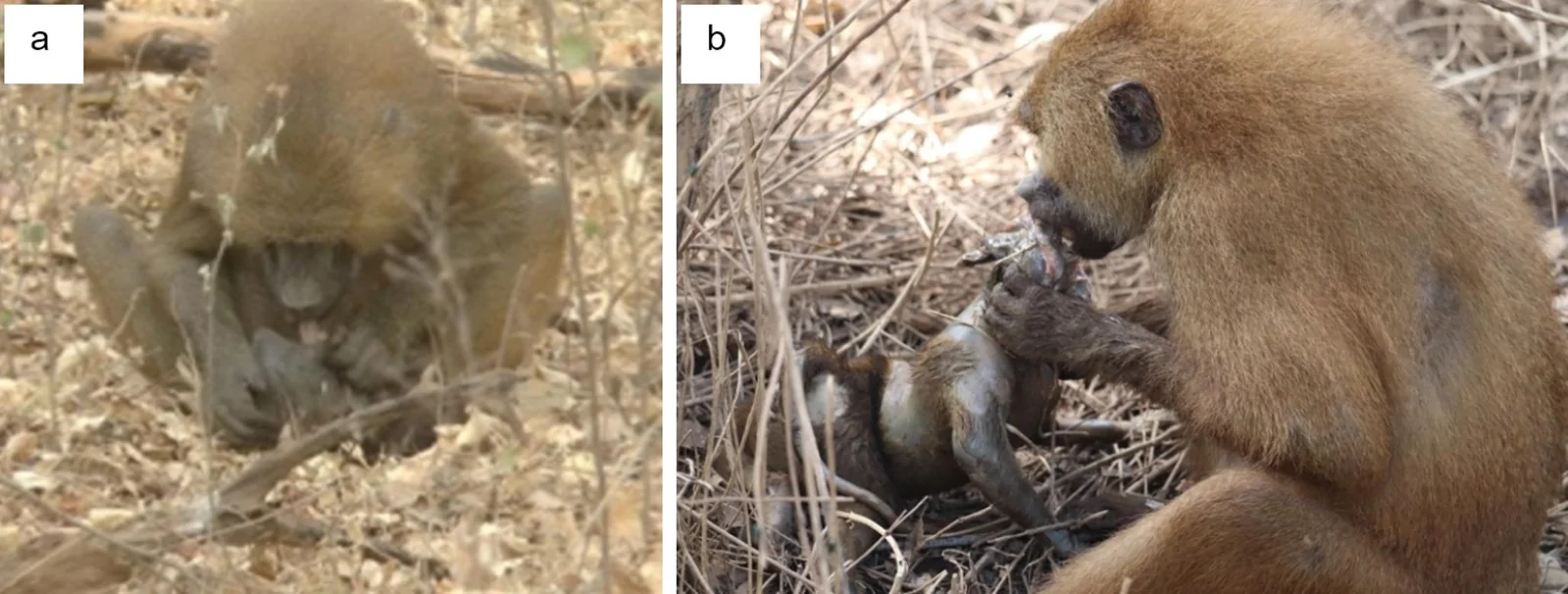
Dead infants being cannibalised by their mothers. In picture (a), the mother is consuming the tongue (case 6); in picture (b), the mother is eating parts of the brain (case 19). Pictures taken by Anaïs Avilés de Diego; Cognitive Ethology Laboratory, German Primate Centre

Corpse-directed behaviours lasted from a few hours to 5 days (Table 2). The median carrying duration was 1 day. Observed behaviours included carrying (Fig. 1), protecting, grooming (Fig. 2), dragging (Fig. S2.1c), and cannibalism (Fig. 3, Fig. S2.3). Carrying occurred in all 22 cases. The corpse was carried in the actor’s arm, either ventrally supporting it (Fig. 1a), holding it in one hand (Fig. 1b), or in the mouth (Fig. 1c). During dragging, the dead infant’s head frequently collided with stones or rocks. Cannibalism was seen on six occasions (27.3%), consisting of eating the corpse’s gums and tongue, sometimes using teeth to strip the flesh (Fig. 3a). Exceptionally, in case 19, the mother also ate other parts (Fig. 3b, Fig. S2.3), including the brain after opening the skull, and flesh of the mandible (see S2 for chronological details of case 19). Neither dragging nor cannibalism was seen when the infant had recently died; instead, these behaviours occurred when the body had started to decompose. In the few cases where dragging and cannibalism occurred the first day the corpse was observed, the advanced state of decomposition suggested that we had missed the beginning of the event.

The cause of death was unknown for all infants. Infanticide seems unlikely as it has never been observed in our population of Guinea baboons; furthermore, pregnant or lactating females have transferred to a new primary male without any subsequent observations of feticide or infanticide. In case 19, the infant had two round puncture wounds at the base of the neck, the precise origin of which was not determined (Fig. S1.1). In case 11, the infant had injuries to the left shoulder and the back (Fig. S1.2), but again we were unable to establish the specific cause of death. On that day, however, the mother walked through a particularly dense thorny area while carrying the infant, and the latter got dragged along the ground for several meters by the primary male of the unit, an unusually rough interaction during which this infant was clearly distressed. Injury from thorns seems unlikely, as the baboons frequently walk through patches of dense thorns with visible resulting wounds (Fig. S1.2).

### Actors of post-mortem behaviours

In 18 out of 22 cases, the only actors of the post-mortem behaviours were the mothers of the dead infants. In one case (case 2), none of the actors was the mother, who had already disappeared or died by the time her dead infant was first spotted. In the remaining three cases (16, 19, and 22), other individuals besides the mother showed post-mortem behaviours with the corpses. These were mostly members from the same unit and party of the dead infant, but also included other individuals previously in the same unit or party as the dead infant’s mother (case 19). In case 16, the dead infant’s older brother touched and carried the corpse, and the adult male of the unit (the likely father of the infant) handled the infant and grunted at it. Case 19 was the longest observed, with many individuals directing behaviours towards the corpse (see supplement section S2), for instance, the dead infant’s older sisters, and another female (from another party) who took pieces of meat from the corpse in full sight of the mother (Fig. S2.4). In none of the 22 cases did any of the actors, including the mother, show any visible signs of distress, such as uttering contact calls or other vocalisations associated with negative affect, such as fear barks or kecks (Maciej et al. 2013).

### Maternal parity and post-mortem behaviour

Eighteen of the 22 dead infants were carried by multiparous mothers; 2 were carried by primiparous mothers, and for one infant, maternal parity was unknown. In the remaining case (case 2), the mother had died, and the female who carried the dead infant was nulliparous, while the second female, who cannibalised it, was of unknown parity.

**Table 2:** Post-mortem behaviours (*N* = 22) towards dead infants in a wild population of Guinea baboons from April 2014 to September 2025

Case no.	Date event (month, year)	Infant ID	Age at death (days)	Infant sex	Female actor’s parity	ID of the actors (mother ID in bold)	Observed duration	Estimated duration	Post-mortem behaviours and age and sex of actors other than the mother
1	07/2015	ATA3	2	M	Multiparous	**ATA**	1 day	2 days^(1)^	Carry
2	08/2015	BMB	37	M	Nulliparous/ Unknown	DPH; XXX	2 days	≥2 day	Carry (DPH); Cannibalism (XXX). DPH: adult female; XXX: unidentified adult female
3	09/2015	APL1	2	NA	Primiparous	**APL**	1 day	1-2 days^(2)^	Carry
4	05/2016	LCY3	2	F	Multiparous	**LCY**	3 days	≥3 day	Carry
5	02/2017	SLY2	Stillbirth	M	Multiparous	**SLY**	1 day	≥1 day	Carry; Protection
6	01/2018	ALI	136	M	Multiparous	**ASA**	1 day	1-2 days^(3)^	Carry; Protection; Cannibalism
7	10/2018	ERE	226	F	Multiparous	**EKA**	1 day	≥1 day	Carry; Grooming
8	02/2019	URS1	Stillbirth	F	Unknown	**URS**	1 day	1 day	Carry; Protection; Grooming
9	02/2019	RCK	89	M	Multiparous	**RAH**	4 days	≥4 days	Carry Protection; Cannibalism
10	05/2019	APX	39	M	Multiparous	**AXL**	1 day	2-3 days^(4)^	Carry; Protection; Grooming
11	03/2020	TKI2	35	F	Multiparous	**TKI**	1 day	≥1 day	Carry; Protection
12	10/2021	BRC	38	M	Multiparous	**BTR**	1 day	≥1 day	Carry
13	02/2022	LSL4	Stillbirth	NA	Multiparous	**LSL**	4 days	≥4 days	Carry
14	02/2023	FRY2	Stillbirth	NA	Multiparous	**FRY**	1 day	≥1 day	Carry; Dragging; Cannibalism
15	03/2023	WHV	119	F	Primiparous	**WDY**	1 day^(5)^	2-3 days^(5)^	Carry; Dragging; Cannibalism
16	04/2024	MMA2	3	M	Multiparous	**MMA** BYE MDM	07:30 h - 10:00 h	150 min	Carry (MMA, MDM); Handle (BYE). BYE: adult male; MDM: juvenile male
17	04/2024	FLP	26	M	Multiparous	**FTA**	8:10 h - 10:00 h	110 min	Carry; Protection; Grooming
18	05/2024	IRN2	Stillbirth	NA	Multiparous	**IRN**	2 days	2 days	Carry; Grooming
19	11/2024	LLM	35*	M	Multiparous	**LSL** VLD SNV LIL LLO WDY TKI MJM SJV QTZ MNT IRN MJV	5 days	5 days	Carry; Dragging; Protection; Grooming; Cannibalism; Meat transfer. *see detailed description in the Supplement for full information. MJM*,* SJV*,* and MJV were unidentified juveniles.*
20	06/2025	OZB	43	M	Multiparous	**ODS**	1 day	≥1 day	Carry
21	07/2025	LSL7	Stillbirth	NA	Multiparous	**LSL**	1 day	1 day	Carry; Dragging
22	09/2025	AGL	203	M	Multiparous	**ASA** MJM	2 days	2 days	Carry (ASA, MJM); Protection (ASA, MJM); Grooming (ASA, MJM). MJM: juvenile male

Age at death is calculated as the number of days from birth until the last day the infant was observed alive. Observed duration indicates the days that the infant corpse was seen. Estimated duration indicates potentially longer durations due to observation gaps between mother and live-infant sightings, infant corpse sightings, and subsequent mother-only sightings

Likely duration and additional comments in our data that suggest a longer duration than the days observed:

^(1)^ The corpse was greyish and had started to smell when it was first seen

^(2)^ The corpse was 1-2 days old when first seen

^(3)^ The corpse was 1-2 days old when first seen; the body was black and rotten

^(4)^ The corpse was in an advanced state of decomposition, mostly skin and bones, with hollow eye sockets

^(5)^ The head and upper body parts of the corpse were missing

* Due to an environmental hazard event (severe flooding), in case 19 there is a gap of 33 days from the date that the infant was seen alive the last time until the corpse was first spotted. Based on the corpse’s fresh appearance, LLM died recently before we saw the corpse. Nevertheless, age at death was calculated as for other cases, despite the potential underestimation for case 19

## Discussion

Over twelve years of observation (2014–2025), we observed post-mortem behaviours directed towards 22 dead infants, around a third of the 64 total cases where we had records of the age at which infants died within 1 year of birth. True rates of post-mortem behaviours may be somewhat higher because we do not have daily records for all animals. As the mean age of infants for whom we observed no post-mortem behaviours was substantially higher (mean 121.2 days) than that of those whose ages we knew (mean 47.1 days), predation or scavenging may account for some cases in which infants went missing, and hence no post-mortem behaviours were observed. With increasing age, infants gradually spend more time away from their mothers (Avilés de Diego et al. 2026) and may face an increasing risk of predation, although we have never directly observed any attacks. Because of this uncertainty about the cause of death and the fate of the corpse, it is difficult to assess the true carrying rate or compare cases where infants were carried or not (Watson and Matsuzawa 2018).

Infant corpse carrying was the predominant post-mortem behaviour, consistent with previous reports in primates, followed by protecting, grooming, and dragging. Cannibalism also occurred. As Guinea baboons are opportunistic predators (Goffe and Fischer 2016; O’Hearn et al. 2025), meat consumption is not unusual. The range of variation in duration of behaviours towards dead infants in this study (< 1 to 5 days) is similar to that reported in other baboon species (e.g., yellow baboons, *Papio cynocephalus*, ca. 3 days: Altmann 1980; chacma baboons, 2–10 days: Carter et al. 2020; Cheney and Seyfarth 2007). Examples of mean and median duration in other primate species are 3.3 days (range, 1–17; SD, 2.7) in Japanese macaques (Sugiyama et al. 2009), 1.8 days (IQR = 1.03–3.59) in eastern chimpanzees (*P. t. schweinfurthii*) (Lonsdorf et al. 2020), respectively. Carter et al. (2020) proposed that the cost of ICC in species that travel long daily distances could lead to shorter dead-infant carrying durations. The long daily travel distances of the Guinea baboons (on average 3.5 km, up to 12 km; Zinner et al. 2021) may indeed promote a relatively early abandonment of dead infants.

The dead infants were carried in the actor’s arm, held across the ventrum for support, held in one hand, or sometimes in the mouth. Guinea baboon mothers do not carry live infants in these ways, nor do they drag them on the ground, unlike some food items such as fruit or prey that might be dragged while moving or travelling. The high occurrence of care-taking behaviours at the onset of the interactions with a corpse and the eventual decline of these behaviours, along with the sequential changes in the way of carrying – from a type typical for a living infant to being carried with one hand, in the mouth, and finally dragged along the ground, all from a careful to a careless manner (Fig. S2.1) – suggest that some infant corpses might initially be perceived as something that requires care-taking but progressively losing this characteristic, finally being perceived as a food item.

Case 19: gave us a partial glimpse into the temporal sequence of post-mortem behaviours with a corpse, and potential changes in mental representation (see supplement section S2). In this case, the mother was carefully interacting with the dead infant until she started cannibalising it. The day the mother started cannibalising the corpse, she still groomed and carried it in a relatively careful manner. Notably, she allowed another female to remove pieces of meat from the corpse (meat transfer), as seen in Guinea baboons after an opportunistic hunting event (Goffe and Fischer 2016; O’Hearn et al. 2025). The maternal behaviour suggests a fluctuating perception of the corpse as both infant and as meat (De Marco et al. 2022; Gonçalves and Biro 2018).

Regarding possible predictors of post-mortem behaviours, our data are informative in two ways, even without statistical testing. Firstly, given that most mothers who showed post-mortem behaviours were multiparous, we provide no support for the idea that dead infant carrying is explainable by the ‘learning to mother’ hypothesis (Warren and Williamson 2004; Watson and Matsuzawa 2018). A recent study on mountain gorilla responses found no effect of parity or season on ICC (Munyawera et al. 2026). However, among non-maternal carriers, nulliparous and primiparous female gorillas were more likely to show ICC than multiparous females, which the authors interpreted as evidence for the hypothesis (Munyawera et al. 2026). Secondly, there was also no evidence in support of the mother-infant bond hypothesis. Mothers were not more likely to carry the corpse of older infants than of newborns (Matsuzawa 1997) or those of intermediate ages (i.e., quadratic relationship of the mother-infant bond with infant age: Fernández-Fueyo et al. 2021).

In which way do observations of ICC inform our understanding of what baboons know about death? Here, our research touches on the philosophical question of whether we can ever know what another species or individual thinks about abstract concepts. In light of this limitation, it seems impossible to *refute* the idea that nonhuman animals lack an understanding of death. Yet the inability to refute this claim does not mean it is correct; that is, it does not justify attributing an understanding of death to the animals (De Marco et al. 2022; but see Monsó and Osuna-Mascaró 2021). From a scientific standpoint, the key question is whether invoking a concept of death is *necessary* to explain the observed behaviours. We argue that it is not.

Regarding whether mothers (or other group members) experience grief, one possible approach is to examine whether they express this emotion. Following Scherer’s component process model (Scherer 2005), emotions involve the appraisal of events and are expressed across multiple response systems, including physiological changes, action tendencies, expressive behaviour, and subjective feeling (Scherer 2005). We infer animals’ emotions from their facial and vocal expressions (Darwin 1872); for instance, in nonhuman primates, fear is often associated with specific facial expressions and vocalisations (e.g., Hesler and Fischer 2007). We did not observe any such emotional expressions in the animals. However, in Barbary macaques living in a large outdoor enclosure, mothers produced protracted contact calls after their dead infant had been removed from the group, compared to remaining silent when they could carry the corpse (Fischer and Hammerschmidt 2002), suggesting that the emotional response was triggered by the separation from the infant, rather than by its death.

Another way to assess maternal perceptions of their infant’s death might be to track their physiological responses. Research on wild chacma baboons revealed that females had elevated faecal glucocorticoid (fGC) levels after loss of a close social partner (Engh et al. 2006). If mothers had a concept of death, elevated faecal glucocorticoid (fGC) levels would be expected both when they have the opportunity to carry their dead infants and when the infant suddenly disappears. Elevated fGC levels after the infant’s disappearance, but not when they can continue to carry the dead body, would point instead to the separation as the cause of distress, not the death.

Interestingly, in multiple macaque and baboon species, we have personally observed that group members sometimes continued to defend the corpse even after it had begun decomposing. If researchers inadvertently came close to the mummified body, they were threatened by other group members, similar to situations when they came too close to a living infant (pers. observations, JF). These anecdotal observations suggest that other group members continue to treat the body as “belonging to them”, irrespective of whether it was dead or alive.

We suggest that the most parsimonious explanation for the observed post-mortem behaviours in Guinea baboons is that they stem from mothers’ innate disposition to form a strong attachment to their infants immediately after birth (Bercovitch 2020). This disposition is strengthened by infantile cues. The lack of infant responses to maternal behaviour and the disintegration of the corpse may contribute to the transition of salience of cues that evoke caretaking to cues associated with food, which ultimately facilitates occurrences of cannibalism. Watson and Matsuzawa (2018) also suggested that cannibalism – once the corpse had started to disintegrate – could indicate a weakening of the mother-infant bond and a transition from regarding the corpse as alive to an object/food item, and that the overlap between caring and cannibalism that occasionally occurs might indicate conflicting impulses (Watson and Matsuzawa 2018). In support of this view, we have also observed grooming of prey after a hunting event, suggesting that fur per se may elicit grooming activity (‘mammalian cues hypothesis’: Reiderman et al. 2024). Taken together, we contend that the Guinea baboons’ behaviour towards dead infants is best explained as cue-driven, rather than concept-driven, and that the observed behaviour does not require an understanding of the irrevocability of death.

## Supplementary Information

Below is the link to the electronic supplementary material.

Supplementary Material

## Data Availability

All data generated or analysed during this study are included in this published article.
