# Supplementary material for: Post-mortem infant-directed behaviours in wild Guinea baboons
Source: Primates. 2026 Jul 26;67(6):681–91. doi: 10.1007/s10329-026-01279-8 (PMC13731491; doi:10.1007/s10329-026-01279-8)
Supplement: Supplementary file 1 [file 10329_2026_1279_MOESM1_ESM.docx]

## Supplementary material for

## “Post-mortem infant-directed behaviours in wild Guinea baboons”

Journal “Primates”

## Anaïs Avilés de Diego^1,2,*^, Federica Dal Pesco^1,2^ & Julia Fischer^1,2^

## ^1^Department for Primate Cognition, Georg-August-Universität, Göttingen, Germany

## ^2^Cognitive Ethology Laboratory, German Primate Centre, Göttingen, Germany

## *Author responsible for correspondence: Anaïs Avilés de Diego

## Address: Cognitive Ethology Laboratory, German Primate Centre, GmbH, Kellnerweg 4, 37077 Göttingen, Germany.

## Telephone number: +49 551 3851-248

## e-mail address: anais.aviles87@gmail.com

## S1. Infant injuries

**Fig. S1.1** Wound at the base of the neck of the dead infant (case 19) (see the two rounded spots). Picture taken by Anaïs Avilés de Diego; Cognitive Ethology Laboratory, German Primate Centre.

**Fig. S1.2** Injured infant with its mother (case 11). Blood is visible on the left side of the infant’s body. Pictures taken by Anaïs Avilés de Diego; Cognitive Ethology Laboratory, German Primate Centre.

## S2. Case 19: Chronology of events LSL-LLM

This text describes the sequence of post-mortem behaviours of case 19 that occurred during five consecutive days, between the mother LSL and her deceased infant LLM. Several individuals interacted with the dead infant besides the mother; see the CRP Simenti work manual (Dal Pesco & Fischer, 2022) for age category definitions and other behaviours mentioned in the supplement besides those that appear in Table 1.

a

b

c

**Fig. S2.1** LLM is initially transported by his mother more carefully, but the carrying changes over the following days to a more careless manner. On day 1 (a), the mother supported the corpse ventrally, on day 2 (b), she held it with one hand, and on day 3 (c), she dragged it on the ground. See the lack of hair in some areas of the body in picture (b), and the lack of hair and body mass in picture (c). Pictures taken by Anaïs Avilés de Diego; Cognitive Ethology Laboratory, German Primate Centre.

**Day 1**

On 04/11/2024, the researchers found the baboon party at 06:47 h. The mother (LSL) was found with her dead black infant male (LLM). LLM still had the black natal coat and light pinkish skin coloration. The death of LLM seemed very recent (a few hours earlier), since the corpse looked very fresh: the skin was still pinkish and did not lack hair; there were no signs of putrefaction: no disfiguration of the face or body, no bloating, nor a foul smell. Moreover, LLM had a wound in the upper part of the back of the head that looked very recent: the blood was bright red and slightly runny; however, it is unclear if the wound was from before death, done post-mortem, or if it was the cause of death. Two black, rounded spots were seen in the base of the neck of the infant (Fig. S1.1). We established the date of death on 04/11/2024, presuming that the infant had died during that same morning. Throughout the day, LSL maintained contact with or kept LLM within close proximity (<1m), and carried LLM carefully, ventrally supporting the corpse with one hand (Fig. S2.1a). LSL also groomed and cradled LLM. LSL started swatting away flies from LLM at some point in the morning, and this continued as the day went on. LSL was protective of the corpse sometimes, mostly of her primary male VLD (subadult male) and SNV (subadult female). The latter grabbed LLM several times and tried to hold him, but LSL firmly held LLM by the arm and leg and pulled him back. SNV groomed LLM on one occasion when LSL was being protective and impeding SNV from getting LLM. Other individuals also interacted with LLM: the older sisters of LLM, LIL (mid-juvenile female) and LLO (small juvenile female), approached and displayed “infant handles”, and LLO also touched, groomed, embraced, and inspected LLM. Infant handles and grunts towards the corpse were observed by WDY (mature adult female), TKI (old adult female), and a MJM (unidentified mid-juvenile male). VLD also displayed infant handle. The corpse was groomed by an unidentified small juvenile of unknown sex and by a SJV (unidentified small juvenile).

**Day 2**

On 05/11/2024, the baboon party was found at 06:56 h. LSL still had the dead infant. The skin of LLM was greyish, darker in the face, and several parts of the body presented absence of hair: most of the head and face, some areas in the chest, abdomen, arms, inner legs, and some patches of the tail (Fig. S2.1b). When LSL was carrying LLM, his head and spine bent back and forth more than the previous day. The body of LLM appeared bloated, and the penis (Fig. S2.2) and the belly button were exceedingly swollen. LLM had a pungent smell (this was the day that the corpse smelled more intensely), and there were many flies around it. The skull bones seemed cracked under the skin (Fig. S2.2), and the upper part of the back of the head had a white-reddish mark, which was most likely the area of the wound of the day before. The two black, rounded spots at the base of the neck were less perceivable.

**Fig. S2.2** Dead infant LLM at the beginning of day 2. The corpse had a white-reddish mark on the upper part of the back of the head, and the skull bones appeared cracked underneath. The swollen penis is visible in the right area of the picture. Picture taken by Anaïs Avilés de Diego; Cognitive Ethology Laboratory, German Primate Centre.

In the early morning, LSL carried LLM ventrally, supporting the corpse with one hand. LSL seemed to carry the LLM less in contact with her torso and less carefully than the day before. LSL groomed LLM occasionally, but much less frequently than the previous day (grooming occurred mostly in the early morning on day 2). Whenever LSL was sitting, she had LLM at her feet and frequently swatted flies away. At around 09:00 h, the number of flies had considerably increased, and so did the frequency with which LSL swatted flies away. During the early morning, QTZ (mid-juvenile male) approached, inspected, sniffed both LLM and LSL’s mouth, grunted, and shortly groomed LLM’s tail. LSL avoided VLD once when he tried to approach, and she was protective of LLM, holding it tightly. MNT (young adult female) also inspected LLM.

At around 10:10 h, LSL was first seen cannibalising LLM: she first ate from the gums and tongue. Shortly after, LSL sat in dense vegetation, almost out of sight of the other baboons and the researchers. In this moment, the cranial cavity of LLM was exposed, and LSL was eating parts of LLM’s brain. Once LSL came out of the dense area, she continued eating from LLM’s brain. At around 10:20 h, LSL was holding a loose piece of skull and eating parts of LLM’s brain adhered to it (Fig. S2.3). Then, LSL dropped the loose piece and directly ate what was left of the brain inside LLM’s skull, while grabbing LLM by the head, and filling up her cheek pouches.

**Fig. S2.3** Dead infant LLM being cannibalised by his mother during the morning of day 2. The mother has a piece of skull in her hands, and she is eating the brain matter attached to it. The dead infant is on the ground, in front of the mother. Picture taken by Anaïs Avilés de Diego; Cognitive Ethology Laboratory, German Primate Centre.

At 10:21 h, the group started moving. LSL carried LLM, and whenever she stopped walking, she continued cannibalising LLM. The transport of LLM was now more careless compared to earlier that day or to the day before, and she held LLM with one hand but did not ventrally support it (Fig. S2.1b), similar to when Guinea baboons carry food or prey. At 10:50 h, LSL carried LLM in her mouth for the first time, but she then returned to carrying LLM in her hands. LSL was also observed dropping LLM carelessly on the ground. Eventually, it was possible to see that the whole LLM’s upper cranial vault and its content were missing, with just some skin left. Throughout the day, LSL continued swatting away flies occasionally.

Once LSL started eating LLM’s brain, some individuals approached her and sniffed and/or looked in her direction. VLD approached LSL-LLM within two meters, and some seconds later, LSL moved several meters away from VLD while guarding LLM. LSL continued eating once she sat down again.

At 10:50 h, LSL and IRN (mature adult female) were sitting next to each other at <1m of proximity, and LSL was eating two loose pieces of LLM’s skull and having LLM’s body next to her. LSL then dropped both these pieces on the ground. Immediately, while LSL was still within 1m, IRN took one of these pieces of bone (Fig. S2.4). IRN was in full view of LSL when this occurred, being the first occurrence observed in Guinea baboons of a mother allowing another individual to remove pieces of meat of her deceased infant (meat transfer: Goffe & Fischer, 2016; O’Hearn et al., 2025).

**Fig. S2.4** Progression of a meat transfer event with the meat of a dead infant. The mature adult female IRN is at the back of the picture, and she is grabbing and manipulating a piece of meat from the dead infant LLM that its mother LSL dropped on the ground. Picture taken by Anaïs Avilés de Diego; Cognitive Ethology Laboratory, German Primate Centre.

For the rest of the day, LSL continued carrying LLM whenever the group moved by holding it in one hand, and sitting down from time to time and dropping LLM at her feet, either in contact or in close proximity (< 1m), eating from her cheek pouches or picking up some pieces of meat from LLM’s head and eating them, and swatting flies away. Eventually, LSL broke LLM’s mandible and ate the meat attached to it. Other individuals remained in the vicinity of the dyad, occasionally approaching and sniffing LSL’s mouth. At 12:02 h, LSL was seen eating a coconut, this being the first time after cannibalism had started that she was eating a food item that was not LLM. From then on, LSL continued eating pieces of LLM, but less frequently than before. The researchers left the baboon party at 12:45 h. By then, LLM had almost no hair on its torso and arms; it was still slightly bloated and smelled strongly.

**Day 3**

The researchers found the baboon party on 06/11/2024 at 08:14 h in an area with very dense vegetation. LSL still had LLM, but she was relatively separated from the main group, about 15m behind. The skin of LLM was dark grey, partially torn, and hairless (Fig. S2.1c). The head was totally unrecognisable, and the bones of the torso seemed to be missing (Fig. S2.1c). LLM had lost most resemblance to an infant. The body of LLM was no longer bloated and had a foul smell, though not as strong as the day before. There were no apparent flies around, but the dense area might have made it difficult to see.

The baboons spent their morning foraging in the dense area. LSL was observed a couple of times with LLM in her hand, but she mostly dragged LLM along the ground (Fig. S2.1c). LSL picked parts of LLM’s belly with her fingers and ate them, but cannibalism occurred less frequently than the day before. On occasions, LSL sat directly on top of LLM. None of the group members showed any behaviour towards the dead infant; LSL was rather away from the group, and no one approached or seemed to have any interest in her and the dead infant (only a MJV – unidentified mid-juvenile – sniffed the corpse). On one occasion, when VLD was passing by, LSL avoided him, but no protective behaviour was observed. The researchers left the baboon party at 12:14 h.

**Day 4**

The researchers did not find the baboon party to which LSL-LLM belonged on 07/11/2024.

**Day 5**

When the researchers found the baboon party again at 06:52 h on 08/11/2024, LSL was still holding on to LLM, who was barely a piece of dry, leathery skin (Fig. S2.5). There were no head or visible body parts anymore, and it had a foul smell. Cannibalism occurred, but with only a few instances being recorded, and since the corpse did not look like an infant anymore, it was very difficult to determine from which part LSL ate. Throughout the day, LSL held LLM firmly, not letting go, for instance, when grooming another individual (Fig. S2.5). On this day, LSL was not seen carrying LLM; she was only dragging him along the ground. No protective behaviour was observed from LSL, and no individual showed any post-mortem behaviour towards LLM. The researchers left the baboon party at 13:02 h.

**Fig. S2.5** LSL holding the dead infant LLM, who is barely a piece of dry, leathery skin, while grooming another individual. Picture taken by Eva Rouselle; Cognitive Ethology Laboratory, German Primate Centre.

**First day without seeing the corpse**

On 09/11/2024, the researchers found the baboon party at 07:02 h, and on this day, LSL did not have the dead infant LLM with her anymore.

## References

Dal Pesco F, Fischer J (2022) CRP Simenti work manual and guides. https://doi.org/10.17605/OSF.IO/AK3W4

Goffe AS, Fischer J (2016) Meat sharing between male and female Guinea baboons (*Papio papio*). Primate Biol 3:1-8. https://doi.org/10.5194/pb-3-1-2016

O’Hearn WJ, Neumann C, Mundry R, Dal Pesco F, Fischer J (2025) Meat transfer patterns reflect the multi-level social system of Guinea baboons. iScience 28(11):113619. https://doi.org/10.1016/j.isci.2025.113619
